# Growth hormone and gonadotropin association: A case report of full-term pregnancy in a patient with panhypopituitarism

**DOI:** 10.5935/1518-0557.20240115

**Published:** 2025

**Authors:** Letícia Quandt, Markus Berger, Laura Gazal Passos, Juliana Trevisan da Rocha, Isabel Cirne Lima de Oliveira Durli, Ivan Sereno Montenegro, Eduardo Pandolfi Passos, Paula Terraciano

**Affiliations:** 1 Medical School- PPGGO/UFRGS; 2 Fertility Center- HMV; 3 Department of Basic Health Sciences - UFCSPA

**Keywords:** hypopituitarism, panhypopituitarism, *in vitro* fertilization, growth hormone, fertility

## Abstract

Hypopituitarism is the inability of the anterior pituitary gland to properly supply the hormone levels. When this disease affects all the hormones produced by the anterior pituitary, it is called panhypopituitarism (PHP). Since pituitary-derived hormones directly influence fertility, often the assisted reproduction techniques are the only option to PHP women have a full-term pregnancy. However, not all patients diagnosed with PHP properly respond to ovulation induction. Thus, a poor response may indicate decreased ovarian reserve or reflect a deficiency in other key components of ovarian function. Here we presented a rare case of a 24-year-old woman diagnosed with PHP and poor response to previous gonadotropin therapy. In our protocol the patient received first growth hormone (GH) replacement for 5 months before starting gonadotropins. When the serum IGF-I (insulin grow factor-I) level normalized, she started ovulation induction with 225 IU/day of human menopausal gonadotropin (hMG). After the ninth day of the cycle, ultrasounds were performed every 2 days to control follicular growth. The puncture of the follicles was performed on the twentieth day of the cycle and a surprising number of 13 oocytes were collected. The oocytes were fertilized by the classical IVF method, resulting in 11 D3 embryos, of which 2 were freshly transferred. Beta hCG hormone levels were determined, and a single fetus pregnancy was confirmed. The birth was by cesarean section at 38 weeks of gestation. Therefore, we conclude that GH/gonadotropin association in ovarian stimulation may improve the follicular recruitment in PHP patients.

## INTRODUCTION

Panhypopituitarism (PHP) is a complex medical condition characterized by the insufficient production and secretion of hormones from the pituitary gland. This disorder affects multiple hormone-producing cells in the anterior pituitary, leading to a deficiency in various hormones that play crucial roles in regulating bodily functions. Among the affected hormones are growth hormone (GH), thyroid-stimulating hormone (TSH), luteinizing hormone (LH), follicle-stimulating hormone (FSH), adrenocorticotropic hormone (ACTH), and prolactin (PRL). Both hypothalamic and pituitary gland disorders can cause PHP. Diseases of the hypothalamus include traumatic brain injury, stroke, tuberculous meningitis, benign tumors that arise in the hypothalamus (such as craniopharyngiomas), and malignant tumors that metastasize to the hypothalamus (like lung and breast cancer). While pituitary gland diseases include agenesis, infections, infarction, pituitary adenomas, pituitary surgery, radiotherapy, and genetic associated conditions (Homburg *et al*., 1990; European and Australian Multicenter Study, 1995).

For women, PHP can have profound implications on their menstrual cycle, ovulation, and fertility. GH has an important effect on the ovary as it not only directly stimulates both steroidogenesis and gametogenesis but also releases gonadotrophins and the insulin-like growth factor-I (IGF-1), which enhance the action of FSH and LH on granulosa cells (Bartke, 1999). The interplay between FSH and LH is essential for the development and release of mature oocytes since inadequate levels of these hormones can disrupt ovulation and lead to irregular menstrual cycles or even amenorrhea. As a result, affected women may experience difficulties in conceiving naturally (Bartke, 1999).

In women with PHP, spontaneous pregnancy, even if rare or exceptional, is associated with a high risk of miscarriages or fetal and maternal mortality (Homburg *et al*., 1990; European and Australian Multicenter Study, 1995; Salle *et al*., 2000). Thus, PHP associated infertility often necessitates medical intervention and assisted reproductive techniques, such as *in vitro* fertilization (IVF), to bypass the hormonal imbalances and help these women conceive. The majority of women diagnosed with PHP can achieve successful ovulation through gonadotropin therapy. However, for those who do not respond well to this treatment, there are limited established options available. Usually, patients who poorly respond to classical ovarian stimulation protocols suffer with several unsuccessful cycles of ovulation induction and/or IVF attempts (Salle *et al*., 2000). In the past decade, based on its ability to modulate the ovarian actions of gonadotropins, GH/IGF-1 replacement therapy has been used for poor responder patients (Zhang *et al.*, 2020). However, publications to date reporting a successful pregnancy outcome in women with PHP receiving GH replacement therapy or GH/gonadotropin association are very rare. These studies support the wide use of IVF techniques for women with PHP and GH to potentiate gonadotropins (Salle *et al*., 2000; Zhang *et al*., 2020; Park *et al*., 2007), but most of them also highlight the variability of protocols and dosages used for hormone replacement, reflecting the lack of a standardized protocol in clinical practice.

In this case report, we presented a rare case of a full-term pregnancy in a young infertile woman diagnosed with PHP and resistance to gonadotropin therapy. The patient undergoes her entire treatment by the Public Health Program in Assisted Reproduction at the General Clinical Hospital, Porto Alegre, Brazil, where she received the support of modern reproductive technologies and a comprehensive medical care.

## CLINICAL CASE

A 24-year-old female previously diagnosed with panhypopituitarism (PHP), was referred to the Gynecology and Obstetrics department at the General Clinical Hospital in Porto Alegre - Rio Grande do Sul, Brazil, for treatment of secondary infertility. She expressed her desire to become pregnant and was included in our Public Health Program in Assisted Reproduction. The patient gave her consent for this report and the Ethical Committee for Human Research of our institution approved this study under the protocol number 2022-0513. The patient had already been under periodic endocrinological follow-up since childhood at the same hospital to treat the PHP condition, which was a consequence of pituitary stalk agenesis. The clinical history includes growth deficiency, primary amenorrhea, hypogonadism, and hypothyroidism. Since adolescence, she was in continuous use of levothyroxine (Euthyrox^®^ 75 mcg/day), testosterone (Deposteron^®^ 25 mg once every 28 days), conjugated natural estrogens (Premarin^®^ 0.3 mg every other day), growth hormone (GH 4 IU/day) and prednisone (Meticorten^®^ 2.5 mg every other day). Menarche was induced by medical treatment at 19 years of age and pubarche and thelarche occurred simultaneously with menarche. At 20 years of age, GH replacement was discontinued. When the patient started at the Assisted Reproduction Program (24-years-old), she was receiving levothyroxine (Euthyrox^®^ - 125 mcg/day), estradiol (Systen^®^ 25 - 1 patch every 2 days), medroxyprogesterone (Provera^®^ 2.5 mg from day 13 to 23 of menstrual cycle), and prednisone 2.5 mg/day.

**Figure 1:**
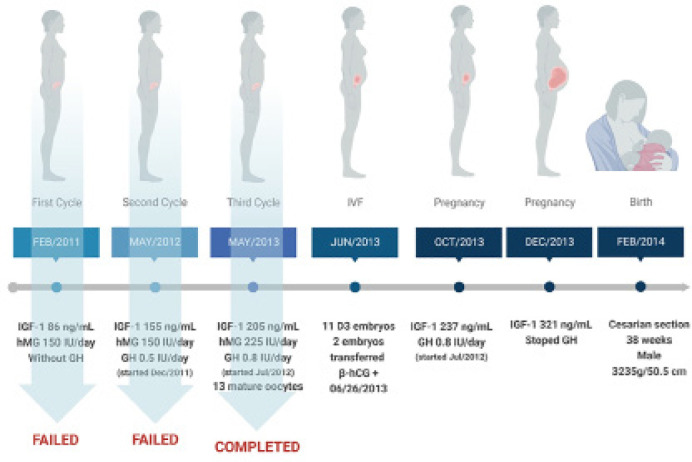
Clinical case.

As shown in Figure 1, the first attempt to ovulation induction was started in February 2011 using the classical protocol with human menopausal gonadotropin (hMG) stimulation (Menopur^®^ 150 IU daily - containing 75 IU of FSH and 75 IU of LH). hMG was administered i.m starting two days after the initial menstrual bleeding, continued during six days, and on the last day, follicular growth and maturation were monitored by ultrasound. Unfortunately, no ovarian response was noticed following this protocol. Thus, we hypothesized that the lack of ovarian response might be related to GH deficiency, since GH replacement was discontinued for 4 years. She did not have testing directly for GH, but the somatotropic deficiency was confirmed by IGF-1 serum determination, indicating a low level of 86 ng/mL (reference range is 114 - 492 ng/mL). Then, in December 2011, the patient started again with GH replacement using a dose of 0.5 IU daily. On May 2012 (5 months after starting GH) her IGF-1 serum levels raised to 155 ng/mL and another attempt to ovulation induction was initiated following the same protocol with hMG 150IU. Despite GH association, this second cycle also failed to induce an effective ovulatory response and was canceled (Fig. 1). For the next cycle we decided to increase the dose of GH to 0.8 IU daily, which improved IGF-1 value to 205 ng/mL. Then the third attempt to ovulation induction was started on May 2013 using this time a hMG dose of 225 IU/day in association with GH (0.8 IU). Follicular growth was controlled by ultrasound and on stimulation day 17 (Table 1), an 18 mm leading follicle was detected and 10,000 IU of hCG (Choriomon M^®^) was given. Thirty-six hours after hCG administration, the follicles were punctured resulting in 13 mature oocytes. Considering that the patient´s partner had healthy seminal parameters (90% total motility and 150 x 10^6^ spermatozoa), the oocyte fertilization was performed by the classical method of IVF. From the total number of 13 inseminated oocytes, 11 D3 embryos were obtained (4 of them with embryonic classification B and 7 with embryonic classification C). Two healthy embryos (classification B) were then freshly transferred to the patient in June 2013 and a β-hCG positive test was confirmed on 06/26/2013. The GH replacement was maintained until December 2013 with IGF-1 levels reaching 321ng/mL. She had an uncomplicated pregnancy, delivering by cesarian section after 38 weeks a normal male child weighing 3,235 g and measuring 50.5 cm.

**Table 1:** Control quality of ovarian follicle and endometrium.

Day	Mature Follicles				Endometrium size (mm)
	Right Ovary		Left Ovary		
	Number	Size (mm)	Number	Size (mm)	
8	6	10	3	10	5
10	3	10	4	10	8
13	3	13	2	12	11
15	4	13-16	4	13-18	8.5
17	6	10-18	5	14-18	10

## DISCUSSION

Our findings in this report suggest that GH plays an important role in ovulation induction mainly through normalization of IGF-1 plasma levels before and during follicular stimulation with gonadotropins. In the case presented here, the patient had pituitary agenesis which caused PHP and a complete GH deficiency. Despite GH replacement during childhood, she stopped the treatment after her menarche and the first attempts to ovulation induction with gonadotropins alone failed. For the next tentative, we decided to start the GH replacement therapy using first a dose of 0.5 IU/day. This dose increased the IGF-1 levels from 86 to 155 ng/mL, but the cycle failed. In the next attempt GH dose was increased to 0.8 IU/day, IGF-1 levels also increased to 205 ng/mL and a positive ovarian response was obtained with gonadotropins resulting in 13 mature oocytes. After a successful IVF procedure, the favorable outcome of pregnancy was achieved.

Previous reports highlighted the potential benefits of GH therapy in combination with gonadotropins for infertile women who respond poorly to gonadotropins alone. Similar to what we observed here, most of the results suggest that GH plays an important role in follicular recruitment at early stages of maturation, and that the normalization of IGF-1 plasma levels before and during follicular stimulation with the use of gonadotropins is crucial (Salle *et al*., 2000; Park *et al*., 2007; Yang *et al*., 2020). In a randomized controlled trial including 16 women with amenorrhea and anovulatory infertility, Homburg *et al*. (1990) showed that combining GH with hMG reduced the required dose of hMG, duration of treatment, and the daily effective dose of gonadotropins. Serum IGF-I significantly increased during treatment with GH but not with placebo. A largest multicentric study concluded that the addition of GH to gonadotropin therapy significantly increased the number of oocytes retrieved, the number of fertilized oocytes, and the number of embryos transferred (European and Australian Multicenter Study, 1995). For these authors the optimal dose of GH was 4 IU/day, and the number of live births increased from 5 in the placebo group (16 patients), to 8 in the GH treatment groups (46 patients) (European and Australian Multicenter Study, 1995). More recently, two other randomized controlled trials presented divergent results (Zafardoust *et al*., 2022; Norman *et al*., 2019). In one of them, the number of oocytes retrieved was higher in the GH group (6.5 *vs*. 4.5, *p*-value=0.001), the number of top-quality day 3 embryos was also higher (2.5 *vs*. 1.5, *p*-value=0.001), and the clinical pregnancy rate was improved (33.3% *vs*. 16.9%, *p*-value=0.04) when compared to a placebo group (Zafardoust *et al*., 2022). The other study reported a greater oocyte retrieval with GH addition, but without a significant improvement in live birth rates following IVF cycle (14.5% in GH group *vs*. 13.7% in placebo) (Norman *et al*., 2019). Additionally, three meta-analysis studies agreed concluding that GH association can improve the main outcomes such as ovarian response, endometrium thickness and live birth rates in poor ovarian responders (Zhang *et al*., 2020; Yang *et al*., 2020; Liu *et al*., 2021).

Regarding PHP patients there are few case reports describing a favorable outcome of pregnancy (Salle *et al.*, 2000; Park *et al.*, 2007). Despite a significant variability of protocols used, dosages and time duration for GH replacement, all the studies reported that GH had an essential role in increasing IGF-1 levels. Similar to what we observed in our patient, these studies also reported favorable outcomes related to ovarian response, oocyte retrieval and pregnancy, just after the IGF-1 serum normalization in PHP patients (Salle *et al*., 2000; Park *et al*., 2007). The molecular mechanism involved is yet unknown. Apparently, IGF-1 does not appear to be mandatory for ovulation and conception since Laron-type dwarfism patients, which have normal GH levels and a complete inability to generate IGF-1, are capable of ovulation and conception. Thus, probably IGF-1 acts more in a synergistic way together with FSH in stimulating follicular maturation. In fact, it is known that IGF-1 can increase the FSH receptor expression in preantral and early antral follicles (Bartke, 1999).

## CONCLUSION

In this case report we described a full-term pregnancy in a poor ovarian responder diagnosed with PHP. Our case confirms the important role of GH/IGF-1 system in follicular recruitment reinforcing the beneficial effects of GH/gonadotropin association for ovarian stimulation in PHP patients. Considering the grater variability of protocols used for GH/gonadotropin replacement therapy, more double-blind randomized studies are needed to generate a standardized protocol in clinical practice.
